# Hyperpolarized NMR Probes for Biological Assays

**DOI:** 10.3390/s140101576

**Published:** 2014-01-16

**Authors:** Sebastian Meier, Pernille R. Jensen, Magnus Karlsson, Mathilde H. Lerche

**Affiliations:** 1 Carlsberg Laboratory, Gamle Carlsberg Vej 10, 1799 Copenhagen V, Denmark; 2 Albeda Research, Gamle Carlsberg Vej 10, 1799 Copenhagen V, Denmark; E-Mails: pernille.rose.jensen@albeda.dk (P.R.J.); magnus.karlsson@albeda.dk.dk (M.K.)

**Keywords:** hyperpolarization, NMR, assays, bioprobes, designed probes, endogenous probes

## Abstract

During the last decade, the development of nuclear spin polarization enhanced (hyperpolarized) molecular probes has opened up new opportunities for studying the inner workings of living cells in real time. The hyperpolarized probes are produced *ex situ*, introduced into biological systems and detected with high sensitivity and contrast against background signals using high resolution NMR spectroscopy. A variety of natural, derivatized and designed hyperpolarized probes has emerged for diverse biological studies including assays of intracellular reaction progression, pathway kinetics, probe uptake and export, pH, redox state, reactive oxygen species, ion concentrations, drug efficacy or oncogenic signaling. These probes are readily used directly under natural conditions in biofluids and are often directly developed and optimized for cellular assays, thus leaving little doubt about their specificity and utility under biologically relevant conditions. Hyperpolarized molecular probes for biological NMR spectroscopy enable the unbiased detection of complex processes by virtue of the high spectral resolution, structural specificity and quantifiability of NMR signals. Here, we provide a survey of strategies used for the selection, design and use of hyperpolarized NMR probes in biological assays, and describe current limitations and developments.

## 1. Introduction

Technological and methodological improvements allow for the study of increasingly complex processes and systems, not least for studying the inner workings of living cells [1,2]. Various detection modalities are used to this end, providing complementary advantages and information for probing and labeling cellular metabolites. For example, several small-molecule and genetically encoded fluorescent probes are under examination for their potential to measure steady-state concentrations, enzyme activities and resulting intracellular reaction kinetics [1,3]. Other methods include IR [4], UV-Vis, luminescence, Raman [5] and NMR spectroscopy as well as destructive detection by mass spectrometry [2]. The choice of appropriate methods requires consideration of the ease of use, commercial availability, sensitivity, biocompatibility, selectivity, spatiotemporal resolution, general applicability, non-invasiveness and quantifiability [1].

NMR spectroscopy is a robust, generally applicable and noninvasive method yielding quantifiable and high-resolution spectroscopic data that can distinguish analytes by resolving individual atomic sites. On the other hand, NMR spectroscopy has shortcomings in terms of sensitivity. In addition, the detection of individual atomic sites usually also leads to complex spectra, as a consequence of the overlap of signals of interest with non-informative cosolute and solvent signals. Isotope enrichment of NMR active atoms with low natural abundance, in particular ^13^C and ^15^N, has been a means to use NMR active probes that are selectively enhanced over background signals by a factor given by their isotope enrichment. NMR spectroscopy is understood from first principles and the interaction between magnetic moments can be used to enhance otherwise weak signals in a controlled manner by transfer of polarization from spins with high magnetic moments (usually protons and electrons) to nuclear spins with lower magnetic moments (e.g., ^13^C and ^15^N). During the last decade, a new generation of nuclear magnetic resonance probes has become popular that affords signal improvements relative to spectral noise and biological backgrounds of at least 3–4 orders of magnitude. This review consecutively covers nuclear spin hyperpolarization, assay designs for hyperpolarized NMR probing, emerging strategies and applications using designed and natural probes, current technological developments and future hopes for NMR assays based on hyperpolarized probes and labels. Several excellent reviews have recently described the development of hyperpolarized contrast agents for functional magnetic resonance imaging [6–9], an application area that is therefore not discussed herein.

## 2. Hyperpolarization of Molecular Probes

**Figure 1.**
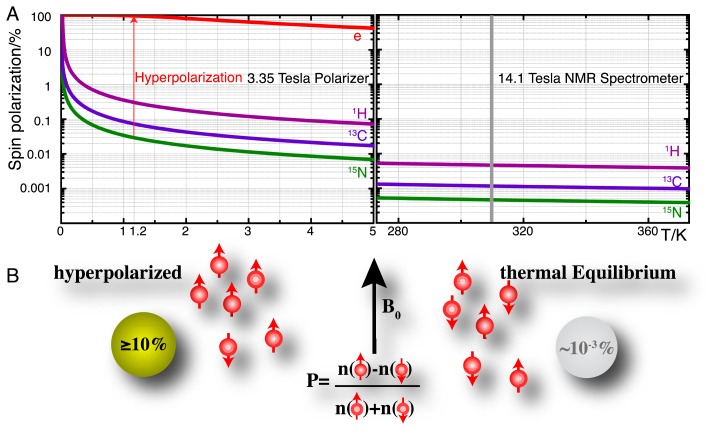
(**A**) Spin polarizations of electrons (“e”), ^1^H, ^13^C and ^15^N nuclei in a 3.35 Tesla DNP polarizer near liquid helium temperature, compared to spin polarizations of ^1^H, ^13^C and ^15^N in a 14.1 Tesla (600 MHz) spectrometer at 273–373 K. An approach to hyperpolarization is the transfer of electron spin polarization to nuclei near 1.2 K prior to dissolution of the hyperpolarized sample in hot aqueous buffer; (**B**) resultant hyperpolarized samples in aqueous solutions achieve spin polarizations P that are ∼3–4 orders of magnitude enhanced relative to the thermal equilibrium polarization in an NMR spectrometer.

High-resolution nuclear magnetic resonance (NMR) spectroscopy has established itself as a principal detection modality in a remarkable variety of disciplines [10–12]. In the life sciences, many of these applications rely on the use of NMR for retrieving molecular information in close to natural environments and intact biofluids, often in order to probe molecular recognition events and biocatalysis. A principal shortcoming of NMR spectroscopy has remained its moderate sensitivity owing to the low equilibrium polarization of nuclear spins as defined for spin-1/2 nuclei by:

(1) $$P_{\text{eq}}=\frac{n^{-}-n^{+}}{n^{-}+n^{+}}≅\tan h\frac{\gamma \hbar B_{0}}{2k_{\text{b}}T}$$

where *n*^−^ and *n*^+^ are the numbers of nuclear spins in the lower and higher energy Zeeman eigenstates, γℏ*B*_0_ is the energy gap between the Zeeman eigenstates and *k*_b_*T* is the thermal energy [13]. The equilibrium nuclear spin determines the fraction of nuclear spins contributing to the detected signal. This fraction remains well below 0.1% for all nuclear spins at currently available NMR spectrometer fields (Figure 1).

Hyperpolarization strategies, such as parahydrogen induced polarization [14], transfer of photon angular momentum to noble gases by optical pumping [15,16], conversion of rotational energy into nuclear polarization upon cooling (Haupt effect) [17,18] and dynamic nuclear polarization (DNP) [19–21] can redistribute the populations of nuclear spin eigenstates far away from equilibrium. DNP is the technique that is most generally applicable in the production of hyperpolarized molecular probes and the principle of these methods is briefly detailed as follows. DNP hinges on the transfer of electron spin polarization from a free radical to nuclear spins by microwave irradiation [19,22,23]. This transfer is best conducted in amorphous samples that assure the homogenous distribution of electron and nuclear spins. DNP is typically performed at low temperatures (<1.5 K) and at high magnetic fields (>3 T) where the electron spin polarization approaches 100% (Figure 1A). Dedicated instruments for DNP under these conditions achieve solid-state polarizations of NMR active nuclei above 10% and are commercially available as so-called “polarizers” (http://www.oxford-instruments.com [24]). The DNP approach to hyperpolarization has gained broad chemical and biological relevance due to a dissolution setup that harvests a hyperpolarized molecular probe by washing the frozen glass of ∼1 K temperature rapidly out of a polarizer with heated buffer [25]. Hyperpolarization losses during this dissolution step can be kept to a minimum and molecular probes with polarizations enhanced by several orders of magnitude can be produced for use in biological assays at ambient temperature and for detection with high-resolution liquid state NMR spectroscopy. A principal limitation of using hyperpolarized molecular probes is the short hyperpolarization lifetime of seconds to a few minutes for non-protonated sites in small molecules.

Hyperpolarized tracers employ a variety of NMR active nuclei with sufficiently slow hyperpolarization loss (determined by the longitudinal T_1_ relaxation time of the nucleus) to perform assays on the minute time scale (Table 1). In practice, these probes combine isotope enrichment with hyperpolarization in order to achieve up to >10^6^ fold signal enhancement over non-informative cellular background signals due to the combined (multiplicative) effect of isotope enrichment and hyperpolarization. The generation and detection of hyperpolarized NMR signal is particularly useful for the nuclei in Table 1 [15,16,25–28], as the low magnetogyric ratios relative to ^1^H leads to small equilibrium polarizations (Figure 1A) and the generation of smaller recorded signal by Faraday induction in the NMR coil (see molar receptivity in Table 1) [29]. At the same time, long relaxation times necessitate long inter-scan recycle delays for some of these nuclei in conventional NMR, thus aggravating their poor utility in conventional NMR detecting nuclear magnetism under conditions of equilibrium spin polarization.

Considering the sensitivity limitation of conventional NMR spectroscopy, it is little surprise that technological and methodological advances resulting in increased sensitivity directly increase the scope of NMR spectroscopy in the study of complex systems. As an example, the ∼4-fold sensitivity gain resulting from cryogenically cooled detection systems has greatly facilitated the in-cell study of recombinant or microinjected isotope-enriched proteins [31,32]. Hyperpolarization approaches yielding 10^3^–10^4^-fold sensitivity gains for molecular probes clearly have significant potential for investigating complex molecular systems such as the inner workings of living cells in a time-resolved and non-invasive manner. The information content of NMR spectroscopic detection is diverse and includes rapid high-resolution spectroscopic readouts of various NMR parameters such as signal frequency, structural motifs and bound nuclei, rotational correlation time and translational diffusion. Spectral information in conventional and hyperpolarized NMR is adaptable by modulating the timing, frequency, power, duration and phase of electromagnetic excitation pulses.

**Figure 2.**
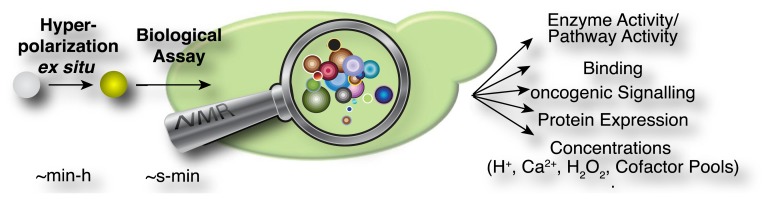
Principle of biological assays using hyperpolarized NMR probes. Hyperpolarization is optimized *ex situ* and the hyperpolarized probe or label is added to a biomolecule, cell extracts or living cells to conduct biological assays for detection inside an NMR spectrometer.

In the current methodological implementations as described above, hyperpolarized probes are produced *ex situ* in a first step, which is specifically designed to optimize signal that is detectable in NMR spectroscopic assays (Figure 2). These assays have been used in diverse experiments for the rapid measurement of steady state concentrations, transporter and enzyme activities and kinetic profiles of cellular reactions. An overview of the hitherto employed probes and assays is provided in Table 2. Predictably, this list may change rapidly as a consequence of the generality of DNP approaches for producing a growing suite of small molecular probes [33], the increasing commercial availability (and popularity) of the technology, improved protocols for probe formulations [33–35] and the recent development of increasingly adaptable platforms for the versatile development of novel probes [36–38].

## 3. Assay Types

**Figure 3.**
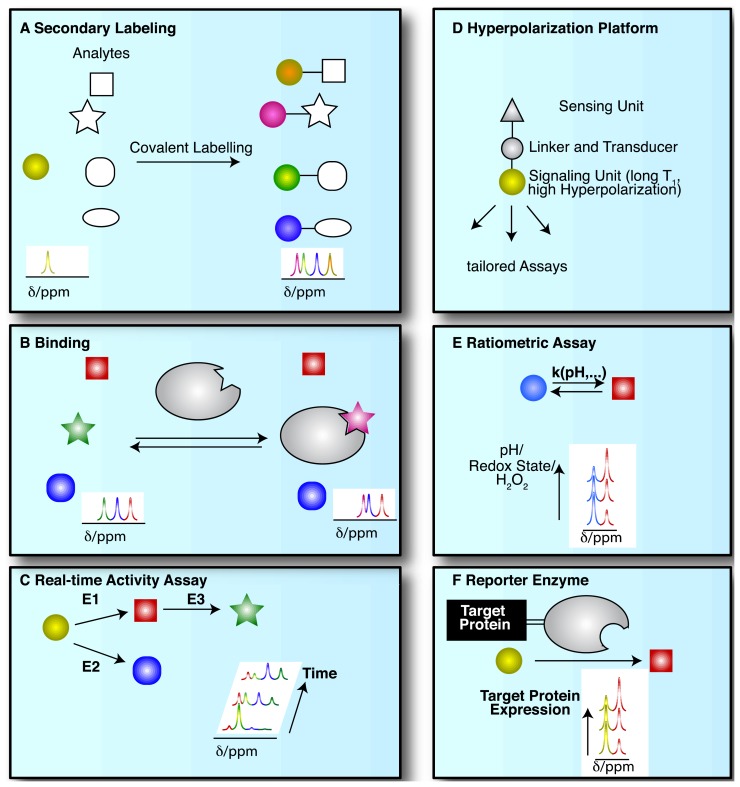
Schematics of different strategies for the use of hyperpolarized labels and probes for NMR spectroscopic biological assays: Hyperpolarized molecules have been used for (**A**) readout by covalent chemical labeling of analytes; (**B**) probing of non-covalent binding; (**C**) the tracking of enzymatic transformations; (**D**) the design of versatile probe platforms; (**E**) ratiometric measurements of physicochemical states and (**F**) interrogating protein expression by probing attached reporter enzymes.

NMR spectroscopic detection of hyperpolarized molecular probes provides rich and adaptable information from versatile assay platforms. Some viable assay types are sketched in Figure 3 with hyperpolarized probes depicted as small colored shapes. Figure 3A indicates an approach taken in the determination of amino acids by “secondary labelling” of amino acids with hyperpolarized [1,1′-^13^C_2_]acetic anhydride [39]. The approach is an adaptation of a chemical derivatization method in conventional NMR at thermal equilibrium. A class of analytes (here amines) is selected from a complex mixture with minimal sample pretreatment by the acetylation with [1,1′-^13^C_2_]acetic anhydride [40]. Upon reaction with different amines, the acetyl label yields resolvable and quantifiable signals for the covalent adducts in thermal and—with improved sensitivity—in hyperpolarized NMR.

NMR spectroscopy has major applications in drug discovery and in particular in hit and lead generation due to the detection of weak binders and the knowledge-based improvement of initial hits [41]. Hyperpolarization of potential binders or mixtures thereof improves assay sensitivity and reduces material demand. As a consequence, the ^13^C-NMR spectroscopic detection of small molecules becomes feasible with good signal-to-noise ratios, thus allowing the observation of binding reactions even at natural isotope abundance of ^13^C, in the absence of solvent (water) signal and with a ∼20 fold larger signal dispersion than ^1^H-NMR [42–44]. Figure 3B sketches the use of hyperpolarized probes for the detection of molecular interactions. Binding reactions are also instructive examples for the versatile readout of processes involving hyperpolarized molecular probes beyond chemical shift changes (Figure 3B). Binding to a macromolecular target changes the molecular environment and thus chemical shift of the hyperpolarized probe. In addition, binding to a macromolecular target affects the rotational tumbling of the tracer and leads to a significant shortening of relaxation times, provoking a shortening of the hyperpolarization lifetime by more than an order of magnitude. In consequence, binders can be identified as signals that exhibit changed chemical shift, line widths or strongly accelerated fading of hyperpolarization. This approach likewise has been used to probe hyperpolarized fluorine in drug molecules at several thousand fold improved sensitivity, reducing the material needed to detect and quantify ligand binding in the strong-, intermediate-, and weak-binding regimes [44]. Yet another readout of probe binding is the transfer of hyperpolarization between competitive binders mediated by the binding pocket of the target [42]. The rapid decay of hyperpolarized binders does not require binding partners that are macromolecular, as demonstrated in the magnetic resonance imaging of benzoic acid binding to cyclodextrins by employing the decreased hyperpolarization lifetime upon binding for contrast generation [45].

In addition to probing drug binding, hyperpolarization was also used in monitoring drug metabolism by discontinuous assays. Here, medication levels in blood plasma were monitored for a anticonvulsant (carbamazepine) that was specifically ^13^C enriched in a position with long hyperpolarization lifetime. Monitoring ^13^C signals rather than ^1^H signals of carbamazepine permitted the resolution and identification of the drug in deproteinized blood plasma with accurate and robust quantifications [46]. Additional contrast relative to background signals can be envisioned by monitoring signals with long hyperpolarization lifetime in backgrounds of faster relaxing signals, for instance by following deuterated ^13^C groups in non-deuterated, rapidly relaxing natural backgrounds.

The most common use of hyperpolarized molecules has been their application in the real-time probing of enzymatic reaction kinetics. In such applications, the chemical conversion of a hyperpolarized organic substrate or metabolite molecule is followed over time, yielding real-time reaction progress curves, also for sequential or parallel reactions (Figure 3C). Once excited to detectable transverse magnetization for detection, hyperpolarization is not recovered. Rather, the transverse component fades with a characteristic transverse relaxation time T_2_ that is shorter than the longitudinal T_1_ time. Hence, progression in binding, transport or chemical reactions is monitored with weak excitation pulses to divide the available hyperpolarized signal for serial, time-resolved readouts [47].

Increased versatility of hyperpolarized probes is recently sought by means of optimized probe design (Figure 3D). Analogous to small fluorescence probe design, hyperpolarized probes have been devised that contain a sensing moiety that is separate from the moiety providing the hyperpolarized NMR signal. Sensing and signaling moieties are then coupled by a transmitter that ensures significant chemical shift changes in the hyperpolarized reporter unit upon events probed by the sensing unit. As the hyperpolarization lifetime is a principal restriction of hyperpolarized NMR probes, the reporter moiety will be selected to provide an atomic site with a hyperpolarization lifetime that is as long as possible. The sensing part of the probe on the other hand is variable and is modified by the analyte of interest.

Hyperpolarized probes have been used to measure concentrations and conditions such as pH, H_2_O_2_ and redox state with ratiometric assays, where these conditions affect reaction rates and equilibrium constants of detectable reactions. Hence, the ratio of signals from two reactants has been used both for rapidly established equilibria and in kinetic experiments (of irreversible reactions, at a defined time point) (Figure 3E).

As a final example, enzymatic conversion of hyperpolarized NMR probes has been suggested for a use analogous to the application of optical reporter enzyme/substrate pairs (e.g., luciferase and luciferin) for monitoring the expression of a target gene in cell biology [37,48,49]. *In vivo* applications of luciferase are limited to observations near the body surface because biological tissues strongly scatter light [37]. Hence, the development of magnetic resonance based reporter protein assays could be advantageous to deep imaging *in vivo*. In one version employing hyperpolarized probes, the gene of a reporter enzyme is fused to the target gene by genetic engineering (Figure 3F). A hyperpolarized substrate of the reporter enzyme then is used to probe the expression of the chimeric target and reporter gene. The hyperpolarized substrate should be a specific substrate of the reporter enzyme and not be converted by endogenous enzymes [37] (Figure 3F). Readout of exogenous enzymatic activities by hyperpolarized NMR has also been suggested for enzymes that are not intracellularly expressed. Such enzymes were for instance targeted to tissues of interest for the activiation of prodrugs to cytotoxic drugs in tumors [48].

## 4. Lifetime of Hyperpolarized NMR Probes

Due to the limited hyperpolarization lifetime even for small molecules, general considerations in the development of hyperpolarized NMR probes primarily concern the optimization of hyperpolarization levels and lifetimes and the choice of probe and assay conditions favoring a rapid readout. The polarization decays with a longitudinal relaxation rate constant R_1_ = 1/T_1_ that is characteristic for the atomic site at a given temperature, magnetic field and molecular tumbling rate. For spin-1/2 nuclei, relaxation is caused by fluctuating magnetic fields at the sites of the nuclear spins. In the absence of paramagnetic relaxation mechanisms [50], relaxation is usually dominated by a dipolar contribution and a chemical shift anisotropy (CSA) contribution [13].

**Figure 4.**
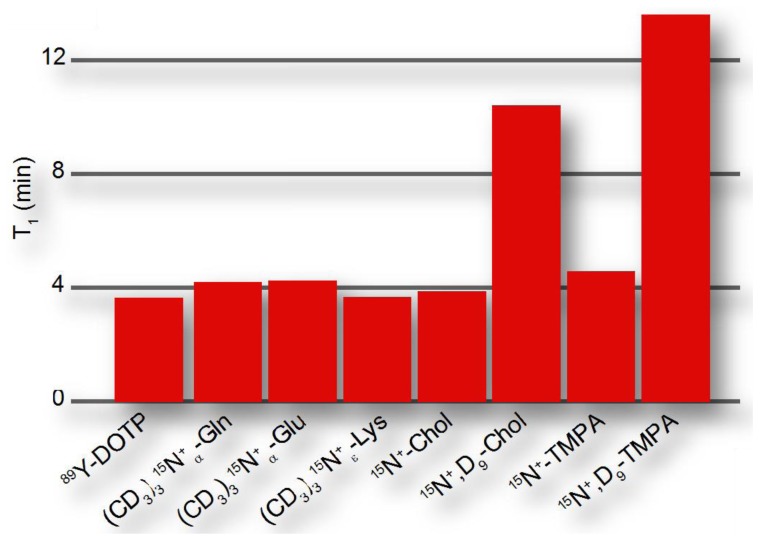
Exponential decay time constants for hyperpolarized reporter groups in various designed probes, reaching up to several minutes in symmetrically substituted, non-protonated sites. The reported time constants were derived at 9.4 T and 25 °C for ^89^Y-DOTP [28], at 14.1 T and 37 °C for permethylated amino acids [51] and at 14.1 T and 30 °C for choline- and TMPA-based probes [38].

The dipolar contribution to longitudinal relaxation of a nucleus X in a molecular probe depends on the nature and distance of nuclear spins as described by the proportionality $R_{\text{d}}\sim \frac{\gamma_{\text{X}}^{2}\gamma_{1\text{H}}^{2}}{r^{6}}$, where γ is the magnetogyric ratio and *r* the distance from the probe nucleus X to the dipolar coupled ^1^H (or other nuclei with large magnetic moment). Hence, hyperpolarized probes will be designed to observe hyperpolarization at a molecular site that is distant from protons, such as quaternary ^13^C and ^15^N atoms [51]. The CSA contribution to longitudinal relaxation is $R_{\text{CSA}}\sim \gamma_{\text{X}}^{2}\Delta \sigma^{2}B_{0}^{2}$, where *B*_0_ is the magnetic field and Δσ is the CSA, which is smaller for symmetrical environments. Hence, hyperpolarized probes preferably contain symmetrical environments around the molecular site serving as the reporter or signaling unit. Bearing these considerations in mind, hyperpolarization moieties have been devised that have exponential decay time constants of up to ∼15 minutes (Figure 4), where hyperpolarized probing is usually considered feasible on a time scale that is 3-5-fold longer than the exponential decay time.

In addition to the direct readout of hyperpolarized signal, magnetization transfer from long T_1_ nuclei storing hyperpolarized magnetization to other, possibly more informative, molecular sites has been reported in various applications [52–54]. As the hyperpolarization lifetime is the Achilles heel of the method in most applications, approaches to manipulate hyperpolarized nuclear spins with pulse sequences to store hyperpolarization in long lived states are currently under vigorous development [55,56].

## 5. Hyperpolarized NMR Probes

Hyperpolarized NMR probes are advantageously categorized into three classes: (i) Non-endogenous probes that are designed for faster delivery or to contain long lived hyperpolarization units for readout of NMR chemical shift changes upon response of an indicator unit to concentrations or conditions in the analyzed system [28,34,36–38]; (ii) Derivatized endogenous molecules, in particular esters [37,57,58], anhydrides [59] and permethylated amino acids [51], that are modified to improve assay properties such as cellular uptake and hyperpolarization lifetimes; and (iii) Endogenous molecules (bicarbonate, vitamin C, metabolites, nutrients) that are used for minimally invasive assays.

Hyperpolarized probes have been designed to obtain beneficial properties relative to natural substrates. In order to enhance probe response during the hyperpolarization timescale, designed hyperpolarized probes have been devised to provide either longer hyperpolarization lifetimes or faster delivery to the site of action, for instance to the intracellular milieu. Permethylation of amino acids, especially with deuterated methyl groups, reduces the proton spin density in the vicinity of amino acid nitrogens and thus decreases relaxation rates of hyperpolarized nitrogen nuclei (Figure 4). When used for perfusion studies, these methylated amino acids do not rapidly enter any metabolic networks [51]. In addition to improving hyperpolarization lifetimes, probes can be derivatized to optimize sample delivery into metabolic networks, for example by esterification of organic acids in order to achieve improved cellular uptake [57,58]. Appropriate balances between hydrophobicity and hydrophilicity should be increasingly considered in optimized probe design particularly for living cell studies, achieving the desired water solubility, membrane permeability and cellular retention of hyperpolarized probes. At the same time, non-natural probes should be biocompatible and bioorthogonal, with the probes exerting no toxic effect in living cells both in their initial or modified forms.

Small natural molecules lend themselves to the direct use as molecular probes if they have non-protonated ^13^C or ^15^N sites. Such sites occur in many metabolites (for instance organic ketones, acids) or can be generated by replacing protons with deuterons, which have much smaller magnetic moments [60,61]. Beyond these considerations, optimization of this class of probes is largely limited to the optimization of hyperpolarization recipes and protocols. As a main benefit, such probes inherently provide biocompatibility if used at near-physiological concentrations. In addition, natural substrates ensure little doubt about the relevance of observed enzyme and pathway activities. The chemical design of small molecule probes, on the other hand, modulates their function relative to the natural substrates [62].

**Figure 5.**
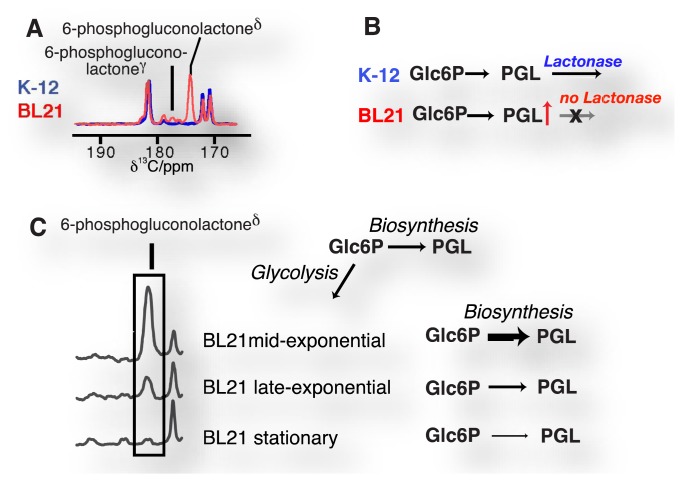
The direct detection of glucose metabolism in Escherichia coli strains shows the accumulation of a lactone intermediate of the pentose phosphate pathway in strain BL21 (**A**,**B**) due to the absence of the lactonase in the BL21 genome, thus affording genomic probing by direct observation of intracellular reaction kinetics; Glc6P = glucose 6-phosphate; PGL = 6-phosphogluconolactone. (**C**) Accumulation of the lactone occurs in a growth phase dependent manner due to reduced usage of a hyperpolarized glucose probe in biosynthetic pathways as cells approach the stationary phase.

Accordingly, enzyme substrates predominate in this class of molecular probes, even if cellular states and concentrations (pH, redox state) are measured. Enzymatic substrates provide the advantage of fairly rapid turnover on the time scale of the hyperpolarization lifetime and of amplified signal through catalytic turnover as compared to binding probes [29]. Observed enzymatic and pathway activities report—amongst others—on qualitative and quantitative changes to reaction usage in disease biology, altered signaling pathways and cellular modifications in treatment, genetic and genomic changes in cells (including transgenic cells) as well as regulation of reaction usage by nutritional states (Figure 5). Besides chemical turnover in enzyme catalyzed reactions, transport processes have been probed by real-time observation with endogenous substrates to determine estimates of the Michaelis-Menten steady-state kinetic constants of the transporters, specifically the maximal velocities and Michaelis constants of glucose, monocarboxylate or urea transporters [86,88,96,99].

**Figure 6.**
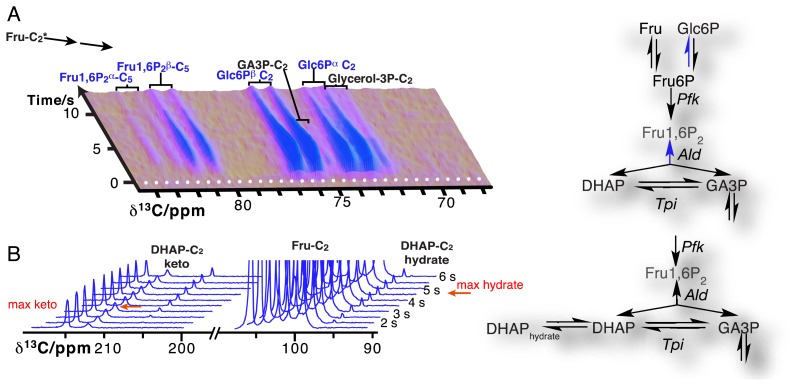
Time-resolved observation of metabolite isomers upon feeding a hyperpolarized [2-^13^C]fructose probe to a *Saccharomyces cerevisiae* cell cultures at time 0: (**A**) Glucose 6-phosphate (Glc6P) and fructose 1,6-bisphosphate (Fru1,6P_2_) C_5_ signals arise from gluconeogenic reactions of the glycolytic substrate. Isomer ratios are consistent with the formation of the isomers from acyclic intermediates; (**B**) real-time observation of dihydroxyaceyone phosphate (DHAP) hydrate formation as an off-pathway glycolytic intermediate (other abbreviations are: GA3P = glyceraldehyde 3-phosphate, *Ald* = aldolase; *Pfk* = phosphofructokinas*e; Tpi* = triose phosphate isomerase).

Due to the resolution of individual atomic sites by high-resolution NMR spectroscopic readout, hyperpolarized NMR probes enable the detection of multiple sequential and parallel reactions. Full kinetic reaction profiles of more than ten metabolites, for instance in microbial glycolysis and fermentation reactions, signify the advantage of using high-resolution readouts to the probing of cellular chemistry [61,85]. In doing so, NMR spectroscopic readouts not only identify a plethora of metabolites, but distinguish their precise molecular forms and the reactivity of these forms. Figure 6A displays the kinetic profiles of sugar phosphate isomer formation by gluconeogenic reactions using a hyperpolarized [2-^13^C]fructose probe as the glycolytic substrate. Isomer ratios underline the gluconeogenic formation of glucose 6-phosphate and fructose 1,6-bisphosphate from acyclic reaction intermediates under thermodynamic reaction control. Using data from the same *in vivo* experiment, Figure 6B indicates the slow formation and decay of hydrated dihydroxyacetonephosphate relative to the on-pathway ketone signal upon using hyperpolarized [2-^13^C]fructose as the probe. Both examples in Figure 6 thus probe the *in vivo* flux of the hyperpolarized signal into off-pathway reactions. On a related note, high spectral resolution also provides the possibility of using several hyperpolarized probes at the same time [100].

## 6. Current Developments and Outlook

Hyperpolarized NMR probes have rapidly shown their biological, biotechnological and recently also clinical [101] potential. The synergistic co-evolution of probe design and probe formulation as well-glassing preparations [33], in conjunction with technical and methodological developments within hyperpolarization and NMR experimentation leave little doubt of an ongoing improvement of hyperpolarized NMR probe technology and applications within the foreseeable future. An increasing selection of metabolite isotopomers—especially ^13^C and ^2^H labeled compounds—will enable more diversified uses of natural (endogenous) hyperpolarized probe molecules for examining biological processes. Diligent choices of probe platforms and the optimization of hyperpolarization conditions will serve to improve probe sensitivity and biocompatibility [102]. Combined optimizations of hyperpolarization lifetime, polarization levels, cellular uptake and retention as well as biocompatibility are yet to be performed for biological assays using hyperpolarized NMR with non-natural probes.

In order to improve assay throughput, approaches employing multiple hyperpolarization chambers [103–105] have been used for multiplexed probe generation. In addition, polarization of ^1^H and subsequent transfer to nuclei with low magnetogyric ratio [106] is a means towards faster hyperpolarization with the DNP method. In addition to using several chambers for probe generation, the use of several chambers for parallel detection in assays, e.g., in multi-chamber bioreactors, will improve assay throughput [107]. The development and use of bioreactors for sustained cell cultures will support assay reproducibility in this context [88,89].

Various NMR methods have been described that provide increased temporal and spatial resolution as well as information content in hyperpolarized probe detection [108–114]. The approaches include modified detection schemes to generate multidimensional spectra from rapid single-scan NMR experiments [54,115–117] or the indirect, amplified detection of signals by saturation transfer methods [86,118]. As mentioned above, a major undertaking is to store hyperpolarization in slowly fading nuclear spin states in order to enhance the utility of hyperpolarized NMR probes in the detection of slower reactions or more pathway steps. Additionally, the assay time window has been extended towards the short end of the time scale by establishing rapid delivery of hyperpolarized substrates into the NMR detection system [119,120]. Resultant time-resolved reaction progression curves over an expanding time scale predictably will increasingly need to be analysed with realistic mathematical models in order to extract quantitative kinetic data [70,71,99,121]. Besides such methodological and technological improvements, ease of use and affordability clearly constitute a major point of concern, especially if hyperpolarized NMR probes are meant to experience routine use in cell biological and clinical assays. While there is room for improvement, hyperpolarized NMR probes already offer a plethora of unique benefits, such as: molecular information and spectral resolution; low background polarization and interference; simultaneous analyte detection; minimal invasiveness especially when using endogenous molecules as probes; the use of non-ionizing electromagnetic radiation with virtually unlimited permeation into tissues and other samples.

Overall, NMR spectroscopy allows minimally invasive observation of complex processes and systems. The development of hyperpolarized probes enables the direct quantitative understanding of such processes and systems in selective assay developed directly for biofluid and cellular settings. In consequence, analytical methods using hyperpolarized NMR help avoid overly optimistic conclusions regarding biological utility and specificity, which can occur with less direct methods that use few selected targets under test tube conditions. Therefore, the use of hyperpolarized probes on complex systems, in conjunction with atomic-resolution NMR detection of probe transport and conversion, has great potential in enhancing our understanding of biological systems.

## Figures and Tables

**Table 1.** Nuclei used in hyperpolarized NMR probes.

**Nucleus**	**Spin I**	**Natural Abundance**	**Molar Receptivity a rel. to^1^H**
^3^He	1	<<0.1%	44.2%
^6^Li	1	7.6%	0.85%
^13^C	1/2	1.1%	1.59%
^15^N	1/2	0.4%	0.10%
^19^F	1/2	100%	83.3%
^29^Si	1/2	4.7%	0.08%
^89^Y	1/2	100%	0.01%
^107^Ag	1/2	51.8%	<0.01%
^109^Ag	1/2	48.2%	0.01%
^129^Xe	1/2	26.4%	2.16%

a NMR signal detection in a coil by Faraday induction is proportional to a factor |γ^3^|I(I+1) where γ is the magnetogyric ratio; the molar receptivity thus describes the NMR signal generated by identical amounts of nuclear isotopes (*i.e.*, enriched to 100%) relative to ^1^H [30].

**Table 2.** Examples of hyperpolarized NMR probing.

**Observable**	**Probe**	**References**
**(i) Designed probes**		

Amino acid concentrations	acetic anhydride	[39]

Binding	^1^H, ^13^C and ^19^F in binders	[42–44]

Drug metabolism	Carbamazepine	[46]

Ca^2+^ concentration	trimethylphenylammonium ubstituted with triacetic acid	[38]

Contrast agent	^6^LiCl	[63]

Enzyme activity	trimethylphenylammonium substituted with methyl ester	[38]

Hocl	*p*-Anisidine	[36]

Hydrogen peroxide	benzoylformic acid trimethylphenylammonium substituted with boronic acid ester	[38,64]

pH	^89^Y-complexes	[28,34]

Protein expression	N-acetyl-L-methionine	[49]

**(ii) Derivatized endogenous probes**		

Enzyme activity	3,5-Difluorobenzoyl-L-glutamic acid (carboxypeptidase prodrug)	[48]

Enzyme activity	ethyl pyruvate	[57]

Perfusion	permethylated amino acids (betains)	[51]

Protein expression	pyruvate derivatives as reporter groups	[37]

**(iii) Endogenous probes**		

Cell permeability, lysis	fumarate metabolism	[65]
	pyruvate diffusion	[66]

Drug efficacy	pyruvate	[67–69]
	fumarate	[65]

Enzyme activities and reaction fluxes		
• Ldh	pyruvate, lactate	[70,71]
• Alt	alanine, pyruvate	[50]
• Bcat	ketoisocaproic acid	[72]
• Glutaminase	glutamine	[73,74]
• Carnitine acetyltransferase, AcetylCoA synthetase	acetate	[75]
• Betaine aldehyde metabolism	choline analog	[76]
• Pyruvate decarboxylase	pyruvate	[77]
• Pyruvate dehydrogenase	pyruvate	[78,79]

Enzyme mechanistic studies	fructose	[61]
	alanine	[80]

Gene expression, gene loss	glucose	[61,81]

Intracellular pH	acetate	[82]
	pyruvate	[83]

Metabolic strategies in different genomes	glucose	[61]

Oncogene signalling	pyruvate	[84]
	ketoisocaproic acid	[72]

Pathway activity, bottlenecks		
• Glycolysis	glucose	[61,85–87]
• Indicator of aerobic glycolysis	[1-^13^C]pyruvate	[71,88,89]
• TCA cycle	[2-^13^C]pyruvate	[90]
• Fatty acid and ketone body metabolism	butyrate	[91]

Redox status	dehydroascorbic acid	[92,93]

Sulfite cytotoxicity	glucose	[94]

Tissue pH	bicarbonate	[95]

Transporter level and activity	
•Glucose transporter	glucose	[86]
•Monocarboxylate transporter	pyruvate	[88,96]
•Urea carrier	urea	[97]

Tumor grading	alanine, pyruvate, lactate	[98]
